# Mechanical and Durability Performance of Coconut Fiber Reinforced Concrete: A State-of-the-Art Review

**DOI:** 10.3390/ma15103601

**Published:** 2022-05-18

**Authors:** Jawad Ahmad, Ali Majdi, Amin Al-Fakih, Ahmed Farouk Deifalla, Fadi Althoey, Mohamed Hechmi El Ouni, Mohammed A. El-Shorbagy

**Affiliations:** 1 Department of Civil Engineering, Swedish College of Engineering, Rawalpindi 47080, Pakistan; 2 Department of Building and Construction Technologies Engineering, Al-Mustaqbal University College, Hillah 51001, Iraq; alimajdi@mustaqbal-college.edu.iq; 3 Interdisciplinary Research Center for Construction and Building Materials, King Fahd University of Petroleum and Minerals, Dhahran 31261, Saudi Arabia; aminali.fakih@kfupm.edu.sa; 4 Structural Engineering Department, Faculty of Engineering and Technology, Future University in Egypt, New Cairo 11845, Egypt; 5 Department of Civil Engineering, College of Engineering, Najran University, Najran 11001, Saudi Arabia; fmalthoey@nu.edu.sa; 6 Department of Civil Engineering, College of Engineering, King Khalid University, P.O. Box 394, Abha 61411, Saudi Arabia; melouni@kku.edu.sa; 7 Applied Mechanics and Systems Research Laboratory, Tunisia Polytechnic School, University of Carthage, La Marsa, Tunis 2078, Tunisia; 8 Department of Mathematics, College of Science and Humanities in Al-Kharj, Prince Sattam bin Abdulaziz University, Al-Kharj 11942, Saudi Arabia; ma.hassan@psau.edu.sa

**Keywords:** natural fibers, physical properties, mechanical properties, durability aspects

## Abstract

The push for sustainability in the construction sector has demanded the use of increasingly renewable resources. These natural fibers are biodegradable and non-toxic, and their mechanical capabilities are superior to those of synthetic fibers in terms of strength and durability. A lot of research recommends coconut fibers as an alternative to synthetic fibers. However, the knowledge is scattered, and no one can easily judge the suitability of coconut fibers in concrete. This paper presents a summary of research progress on coconut fiber (natural fibers) reinforced concrete. The effects of coconut fibers on the properties of concrete are reviewed. Factors affecting the fresh, hardened, and durability properties of concrete reinforced with coconut fiber are discussed. Results indicate that coconut fiber improved the mechanical performance of concrete due to crack prevention, similar to the synthetic fibers but decreased the flowability of concrete. However, coconut fibers improved flexure strength more effectively than compressive strength. Furthermore, improvement in some durability performance was also observed, but less information is available in this regard. Moreover, the optimum dose is an important parameter for high-strength concrete. The majority of researchers indicate that 3.0% coconut fiber is the optimum dose. The overall study demonstrates that coconut fibers have the creditability to be used in concrete instead of synthetic fibers.

## 1. Introduction 

Conventional concrete (CC) is generally robust in compression but not in tension. Steel bars are often employed when tensile stresses are created or tension zones are discovered to overcome this deficit in traditional concrete, which is commonly referred to as reinforced cement concrete (RCC). Fibers are added to concrete to increase its inherent tensile strength, resulting in a specific form of concrete known as fiber reinforced concrete (FRC) [1,2,3,4]. It is desirable that beams be ductile rather than brittle [5].

Besides secondary cementitious materials, which help in ecofriendly concrete [6,7,8], natural fibers have emerged as one of the most popular reinforcing materials in terms of sustainability and biodegradability [9], Non-toxic, and environment-friendly [10], features that are particularly beneficial for the manufacture of biocomposites natural fibers, on the other hand, help to reduce CO_2_ emissions into the surrounding environment as well. Biocomposites are becoming more popular as appealing products in a variety of industries, including automotive, aviation, packaging, construction, architectural, and biomedical [11]. Furthermore, natural fibers can be found all over the world, which are cheaper than artificial fibers, have more stiffness, and can be recycled [12]. Coir is a popular natural fiber derived from the husks of mature coconut fruits and is used to make high-strength and long-lasting products [13]. Due to the many advantages of natural fibers, including their widespread availability, biodegradability, lightweight, cheap cost, and simplicity of manufacture, natural fiber-based biocomposites have largely displaced synthetic plastics in a range of applications [14]. Numerous researchers have suggested a variety of natural fiber composites for use in a variety of technical applications [9,15,16,17]. Many countries across the globe, particularly in tropical and subtropical regions, cultivate coconuts, which play an important role in economic growth. Coir fibers from over fifty billion coconuts are gathered across the globe, according to recent research [18].

A growing number of academics and scientists have been interested in using natural fibers as an alternative to conventional glass and carbon fibers as a reinforcement in polymer composites in the last several decades [19]. Among the natural fibers that may be found in clothing are flax, jute, kenaf, coir, banana husk, and henequen [20,21,22]. Cost, density, specific tensile properties, non-abrasive to equipment and skin, reduced energy consumption, less health risk, renewability, recyclability, and biodegradability are all advantages of natural fibers over man-made glass and carbon fibers. Fereshteh incompatibility of hydrophilic natural fibers with hydrophobic thermoplastic matrices is a known disadvantage of natural fiber/polymer composites. This leads to unwanted composite characteristics. To promote fiber-matrix adhesion, the fiber surface must be chemically modified [23]. Coconut fiber has been the subject of several researchers who have sought to enhance the performance of concrete by using it as a fiber reinforcing material [24,25,26].

Natura fibers have extremely good compatibility with a variety of thermoplastics, thermosetting polymers, and cementitious materials due to their lower density, better thermal insulation properties, mechanical properties, lower prices, unlimited availability, nontoxic approaches, and problem-free disposals, among other characteristics. Despite the fact that natural fibers have been extensively investigated in terms of their thermal, mechanical, and morphological characteristics, little is known about their compressive qualities. As a result, this study discusses the chemical, physical, workability, mechanical, and durability properties of coconut fiber reinforced concrete, as well as their application. Coconut fiber is classified into the following four types: bristle coil, buffering coil, brown fibers, and white fibers. Bristle coil is the most common form of coconut fiber. The brown fiber is generated from mature coconut, and it is typically highly strong, thick, and has great abrasion resistance, making it the most favored and most widely used fiber on the planet [27]. Unlike the brown fiber, which comes from mature coconut, the white fiber comes from immature coconut and is often not as strong as the brown fiber. The fiber has a large amount of lignin material and a low amount of cellulose, which makes the fibers versatile, solid, and strong [28]. Coconut fibers are available in the following two varieties: treated and untreated. Either by soaking the fibers in hot water or by soaking them in chemical solutions, they may be cured [29]. According to a study, when treated natural fibers are incorporated into the concrete using the compression molding method with a 10 percent fiber weight, the resulting concrete has a very high tensile strength when compared to composites that have untreated fibers incorporated into them. Tensile and fatigue tests were performed on the treated natural fibers to confirm this claim. The removal of lignin, pectin, wax, and hemicellulose from the fiber surface resulted in the elimination of parenchyma cells, which increased the contact area of the exposition of fibrils and globular marks. As a result, the roughness of the fibers increased, which in turn increased the adhesion between the matrix and the fibers [30]. In addition, coconut fibers have low heat conductivity while being strong and stiff, so they enhance the tensile, flexural, and compressive strengths of concrete while at the same time reducing the weight of the concrete [31]. 

Many variables may impact the performance of natural fiber-reinforced composites, including the fiber type used and the amount of fiber used. Aside from the hydrophilic nature of the fiber, the characteristics of natural fiber-reinforced composites may be altered by the quantity of fiber used in the composite and the amount of filler used. In general, substantial fiber content is essential for composites to function well in order to obtain high performance. The optimum is also important for the better performance of concrete. The effect of fiber content on the properties of natural fiber-reinforced composites is particularly significant. A lot of researchers focus on coconut fiber instead of steel fibers. As the steel fiber is costly as well as thermal expansion and corrosion problems. However, the knowledge of coconut fiber in concrete is scattered, and no one can easily judge the importance of coconut fiber in concrete. Therefore, this review focuses on the physical properties of coconut fibers, fresh properties, and mechanical and durability aspects of concrete reinforced with coconut fiber. A successful project will also give the idea to a new researcher to choose and apply coconut fiber in concrete. 

## 2. Physical Properties of Coconut Fibers 

**Figure 1:**
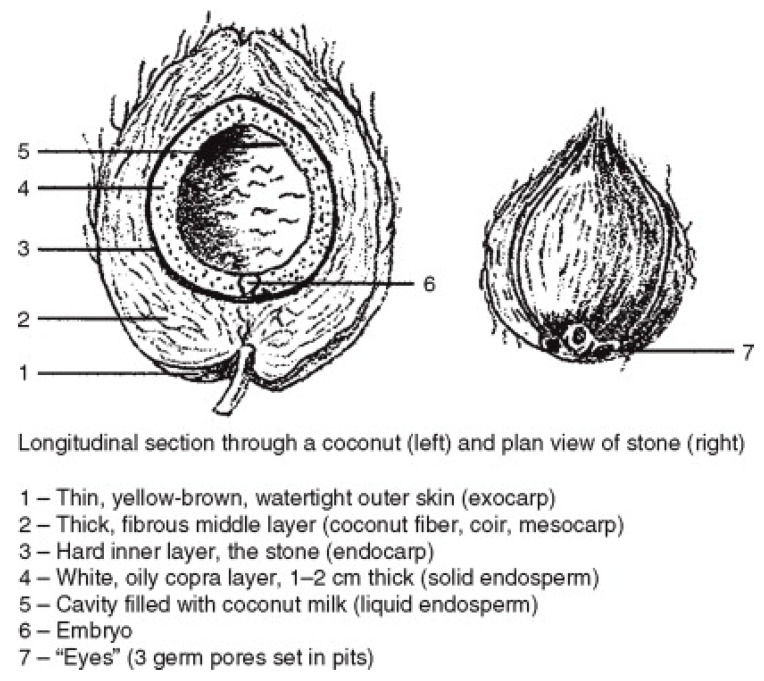
Longitudinal section of coconut and plan view of stone.

Coconut fiber, also known as coir, is derived from the fibrous husk of the coconut plant and is used in the production of coir. This is the thick fibrous middle layer of the coconut, which is shown in Figure 1 as a thick fibrous middle layer. Coconut shells are sliced in half and retted to remove the fibers from the meat. It is necessary to bury the coconut shells in damp soil during the retting process in order to enable the microbial breakdown of the softer tissues to occur. After that, the shells are smashed and rinsed in order to easily extract the coir fibers. By the way, the “stone” is the term used to describe the hard inner layer [32].

Coconut fibers are mostly brown in color with varying lengths and diameters. Moreover, other properties such as tensile strength and modulus of elasticity vary depending on the source and usage. A different researcher reported different physical properties of coconut fiber. The details of the various physical properties of fibers as per past researchers are given in Table 1.

## 3. Fresh Properties 

Concrete workability is a term that relates to how easily mixed concrete can be placed, compacted, and finished while retaining its homogeneity to the greatest extent possible. Unworkable concrete is one that cannot be easily worked. In unworkable, the cement paste is not sufficiently lubricated and it does not adhere to the aggregates correctly, resulting in significant aggregate segregation. Maintaining the homogeneity of an unworkable concrete mix is very difficult, and compaction of concrete requires a significant amount of work, which has a negative impact on the mechanical and durability performance of concrete.

**Figure 2:**
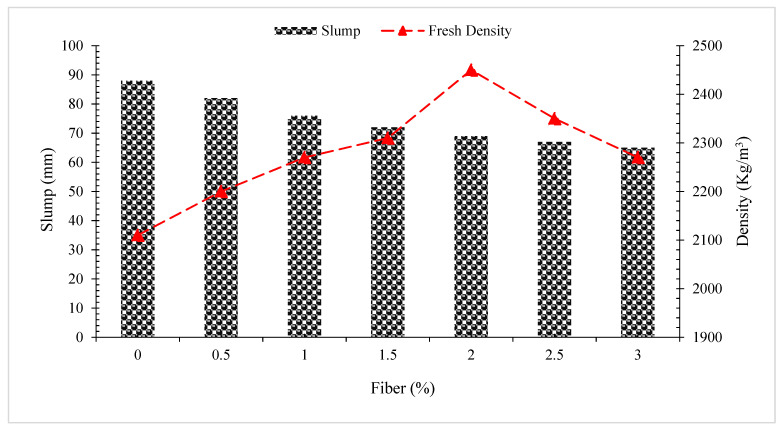
Slump and fresh density of concrete with coconut fibers.

According to one study, the value of slump flow decreased when the dose of coconut fibers was raised. The maximum slump flow was reached at zero percent addition (control concrete) while the lowest slump flow was recorded at three percent addition of coconut fibers, as indicated in Figure 2. The increased surface area of coconut fibers requires more water to cover, resulting in less free water for oiling. Moreover, coconut fibers increased internal friction among concrete elements, necessitating more cement paste [38]. Adding 0.25 percent coir fiber (by weight of aggregate) reduced slump to 50 mm. The slumps of the following variants indicate decreasing values with increasing coir fiber content. This propensity is due to the coir fibers’ surface shape and physical qualities [39]. According to one study, coir fibers have hydrophilic surfaces and hence repel water [26]. In a similar manner, several studies have shown that the slump value decreases when coconut fiber is included [40,41,42].

As illustrated in Figure 2, according to one study, fresh density rose as the proportion of coconut fiber increased up to 2.0 percent, after which it decreased when compared to reference concrete. Coconut fiber at a dose of 2.0 percent exhibits the highest fresh density when compared to the reference concrete (0 percent addition of coconut fibers). However, the fresh density was lowered with the additional addition of coconut fibers, with a minimum fresh density of 3.0 percent when compared to other coconut fiber reinforced concrete. The increase in fresh density of concrete reinforced with coconut fibers is related to crack prevention since coconut fiber reinforced concrete has fewer plastic shrinkage voids and produces denser concrete. However, with the 4% addition of coconut fibers, compaction becomes problematic, resulting in porous concrete and lower fresh density. A study claim that adding 1.5 percent fibers by volume to concrete increases density by 15% as compared to the reference concrete [43]. In contrast, one study found that when the fiber content of the specimens increased, the density of the specimens dropped. Due to the fact that fibers are light, their addition to concrete causes cavities in the matrix, which reduces the density of the concrete. As a consequence of the inclusion of low-density coconut fibers, a phenomenon is known as the “filled void effect” occurs, which decreases the density of the concrete when compared to plain concrete. 

**Figure 3:**
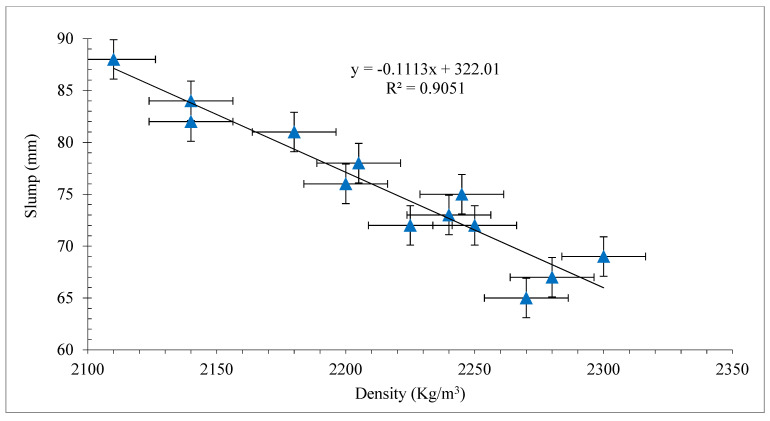
Correlation between slump and fresh density with coconut fibers.

Density is an important factor that influences the flowability of concrete. A lack of workability leads to voids in occupied space and reduced density. Figure 3 shows the link between fresh concrete density and workability with increasing percentages of polypropylene fibers. Workability and concrete density of fiber reinforced concrete have a good association (R^2^ > 90%).

## 4. Treatment of Coconut Fibers 

To investigate the durability of coconut fiber, several treatments were carried out on the material. For the first minute, each coconut fiber was soaked in an adherent solution (deionized water or natural latex) to ensure that it adhered to the other fibers. During this process, the adherent solution surrounds the coconut fiber, forming bonding layers between the two materials. A coating agent was then applied, which consisted of pozzolanic materials (silica fume or metakaolin). Pozzolans are attracted to the coconut fiber by the adhering solution in which they are dissolved. The production of “chicken fingers” is comparable to the process used in the development of this novel medicine [44]. Table 2 shows the details of the surface treatment of coconut fibers.

Using a latex polymer film and a pozzolan layer, the coconut fiber treatment was created to increase the flexural strength and durability of cement-based composites. The performance of the sample treated with silica fume and natural latex was 42.2 percent better than the performance of the sample without any treatment. When fiber samples were subjected to degradation tests, the mass conservation rate increased as a consequence of this treatment (silica fume and natural). This treatment resulted in an enhancement in the retention of the fiber structure against the degradation process, according to the microstructural examinations of the treated fibers isolated from CF. This treatment (silica fume and natural) has the potential to be a viable alternative to the use of coconut fibers in the creation of novel cementitious composite materials that have appropriate performance and long-term durability. The compressive and flexural strengths of the structures increased by up to 13 percent and 9 percent, respectively, according to the testing data. However, in terms of durability, the chloride penetration, intrinsic permeability, and carbonation depth increased with CF. The authors propose that the coconut fiber could be treated prior to being used in concrete to ensure that it is protected against deterioration [37]. Two approaches were employed to enhance the durability of cementitious composites by using vegetable fibers. Both treatments are applied in this investigation, namely, a surface treatment of the coconut fibers with a polymeric film of natural latex mixed with a pozzolan layer and a pozzolan layer alone. Based on the findings of this research, the treatment used to treat the fibers generates local pozzolanic reactions that affect both the cementitious matrix around them as well as the surface of the fiber, therefore preventing alkaline attack and mineralization of the fibers [45]. Coconut fibers were immersed in NaOH solutions with concentrations ranging from 2 to 10% for four weeks. The authors discovered that the tensile strength reduced as the concentration of NaOH increased, which they attributed to the fibers becoming more fragile [46]. 

## 5. Mechanical Properties

### 5.1. Compressive Strength 

Compressive strength is a material’s or structure’s capacity to bear loads without cracking or deflection. Compression shrinks a material’s size. Concrete’s compressive strength gives an indication of the concrete’s properties. This single test determines whether or not concrete was correctly performed. In commercial and industrial construction, concrete’s compressive strength ranges from 15 MPa (2200 psi) to 30 MPa (4400 psi). Compressive strength is tested on a cube or a cylinder. The American Society for Testing Materials established ASTM C39/C39M [47] for compressive strength testing of cylindrical concrete specimens.

**Figure 4:**
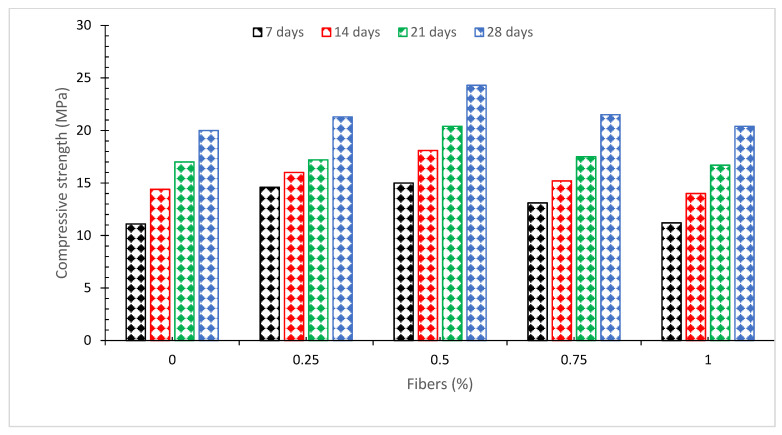
Compressive strength [48].

Figure 4 depicts the compressive strength of concrete with various doses of coconut fiber (length 60 mm and diameter 0.75 mm) ranging from 0% to 1% in 0.25% increments for concrete mix 1:2:4 (M15) with water to cement ratio of 0.58. The compressive strength of concrete rose to 0.5 percent with the addition of coconut fiber and then steadily dropped, with the lowest compressive strength at 1 percent after the addition of coconut fiber and the highest compressive strength at 0.5 percent. 

Research also found that fibers boosted concrete’s compressive strength up to a certain point before decreasing owing to a lack of workability [38]. Even at a larger dosage, the compressive strength of the concrete is lower than that of the reference concrete. The fiber reinforcement’s confinement on the specimen has a favorable impact on compressive strength. Compression results in lateral expansion, which is limited by the coconut fibers (CF), resulting in increased compressive strength. Because of their strength, the fibers can sustain strain and shear [38]. Compaction becomes problematic at larger doses (more than 2.0%) owing to a lack of workability, resulting in decreased strength. A study reported that in comparison to reference concrete, 1.5 percent of the fibers enhanced compressive strength by over 15% [43]. At 1.0 percent by volume, fibers significantly improve the mechanical performance of concrete at both the initial and later ages. The greatest 28-day strength increase was found to be 29.15 percent [49]. As a result, coconut fiber has an ideal limit. The experiments indicate that the best dosage of coconut fiber for strength is 2.0% by weight of cement [26]. A study indicates that the optimal quantity of coir fiber in concrete is 0.25 percent, which results in a 19% increase in 28-day compressive strength [39]. However, the optimum dose of fibers varies depending on the type of fiber, the physical aspects such as length and diameter, as well as the concrete mix design and the water-to-binder ratio. A study reported that in coconut fibers of 50 mm and 75 mm long, the compressive strength decreases with the increase in fiber content. The decrease in compressive strength could be attributed to the decreased workability of fresh concrete caused by the increased content and length of fibers, as well as the lack of proper compaction during specimen casting, resulting in the formation of air voids. It might be possible due to the dilution of the cement matrix/hardened cement paste caused by the addition of fibers [50]. 

**Figure 5:**
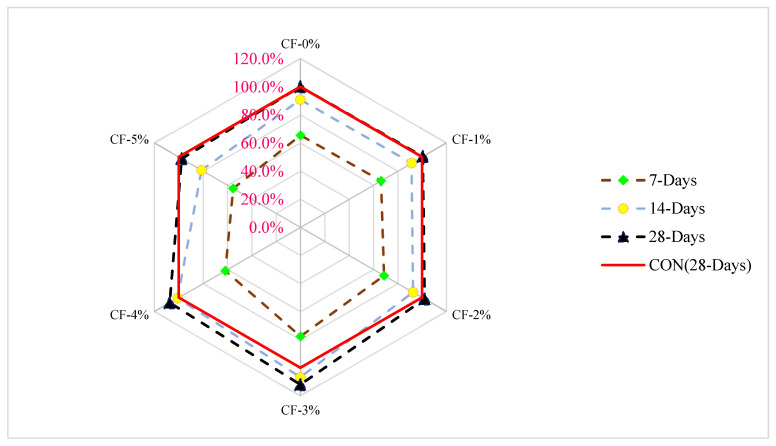
Relative analysis of compressive strength [24].

Figure 5 shows a relative analysis of compressive strength with varying doses of fiber on different days of curing. The compressive strength of control concrete at 28 days of curing was considered as a reference mix, from which the compressive strength of other doses of coconut fiber (CF) was compared at different days of curing. The optimum dose of CF (3%) was considered for a comparison analysis. At 7 days of curing, compressive strength is 33% less than compared to reference compressive strength (28 days of controlled concrete compressive strength at 3% addition of CF. At 3% addition of CF, the compressive strength is 6% more than the reference concrete compressive strength at 14 days of curing. At a similar dose of CF (3%), compressive strength is 12% more than the reference concrete. It can be observed that coconut fiber does not considerably improve the compressive strength of concrete. Similarly, a study also reported that fiber improved the tensile strength of concrete more effectively than compressive strength [38]. 

### 5.2. Split Tensile Strength 

Splitting tensile strength refers to the stresses generated when compressive loads are applied using compressive testing equipment in such a manner that a concrete cylindrical specimen splits vertically in half. It is referred to as the indirect approach for determining the tensile strength of concrete. The direct technique is not practicable due to insufficient grip on the cylindrical sample and the eccentric force. As a result, the direct tensile test is not the standard procedure. At the stipulated duration of curing, concrete tensile strength was discovered according to ASTM C496-71 [51] for a typical cylindrical sample of size 150 (mm) in diameter and 300 (mm) in length.

**Figure 6:**
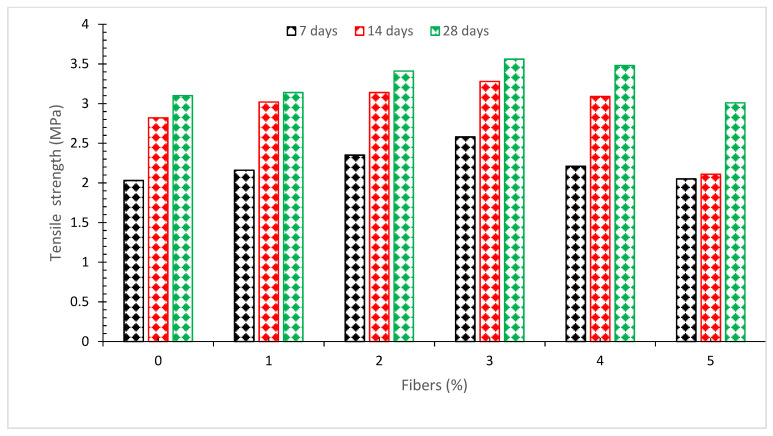
Tensile strength [24].

Figure 6 shows that the greatest concrete tensile capacity was recorded at a 3.0 percent addition of CF as compared to the control (0 percent fibers) after specified days of curing [24]. 

According to the results of the research, the split tensile strength is 40 percent greater than the reference mix when just 2.0 percent of the fibers are added. The fact that fibers enhance tensile strength more than compressive strength is also mentioned [52]. When coconut fiber is added at a concentration of 3.0 percent, the lack of workability results in a gradual decrease in split tensile strength. Because coconut fibers inhibit fracture formation and increase tensile strength, they improve the flexibility of concrete and increase its tensile strength. Fiber-reinforced concrete performs better than normal concrete in terms of strength and durability. Cracks are stopped rather than prevented by CF. Notably, CF has a greater effect on tensile strength than it does on compressive strength. Fibers have been shown to improve the behavior of post-cracked concrete [53]. Furthermore, fibers having from 0.5 to 2.0 percent by volume have a much greater influence on the tensile strength of concrete than fibers containing less than 0.5 percent by volume [54]. It was discovered that increasing the fiber content from 0 to 20 percent by weight enhanced the tensile strength by almost thrice [55]. Coconut fibers, with a tensile strength of 21.51 MPa, are the strongest natural fibers. It has the ability to sustain forces that are four to six times larger than those experienced by other natural fibers. A large number of studies have looked at the usage of coconut fibers for a range of different reasons. Some characteristics are significantly different; for example, the diameter of the coconut fibers is nearly the same and the levels of tensile strength are somewhat different. For example, the fibroblasts of various individual cells were dependent on the type of plant, its location, and puberty, among other factors [56]. In the study, it was discovered that treatment of the fiber decreased its tensile strength. The lignin, pectin, fatty acid, and cellulose levels are reduced as a result of the treatment approach. Tensile stresses at the failure of fibers were elevated by 18 percent and 51 percent, respectively, as a result of chemical and physical treatments. Thus, treatment has been shown to improve the ductility of fibers [57]. According to one study, increasing the amount of coconut fiber in a material may increase the tensile split strength by as much as 5 percent. When the fiber content increases over this threshold, a reduction in tensile stress is seen. It is possible for concrete to fail under tension due to disturbances in the presence of atoms and molecules in the concrete mix design. By adding them to the mix, they act as a connection that helps to hold the fibers together [56]. In contrast, the inclusion of coir fibers reduces the split tensile strength of concrete [39]. 

**Figure 7:**
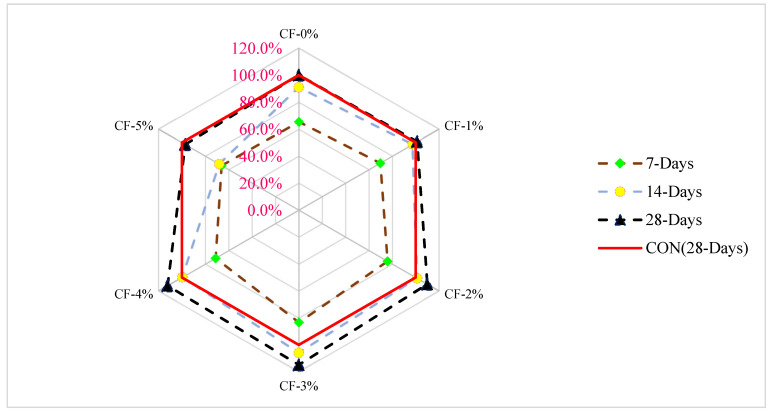
Relative analysis of tensile strength [24].

In the relative analysis of concrete shown in Figure 7, the 28-day tensile strength of the control mix is used as the reference mix, and then other mixes with varying percentages of coconut fiber are compared to the reference mix. When 3.0 percent of coconut fiber is added, split tensile strength is about 17 percent lower than reference concrete at 7 days of curing. At 14 days of curing, the tensile capacity of concrete is only 5 percent more than that of reference concrete when coconut fiber is added 3.0 percent. At the same dose of 3% of coconut fibers, the tensile capacity of the concrete is 15 percent greater than the reference concrete after 28 days of curing.

**Figure 8:**
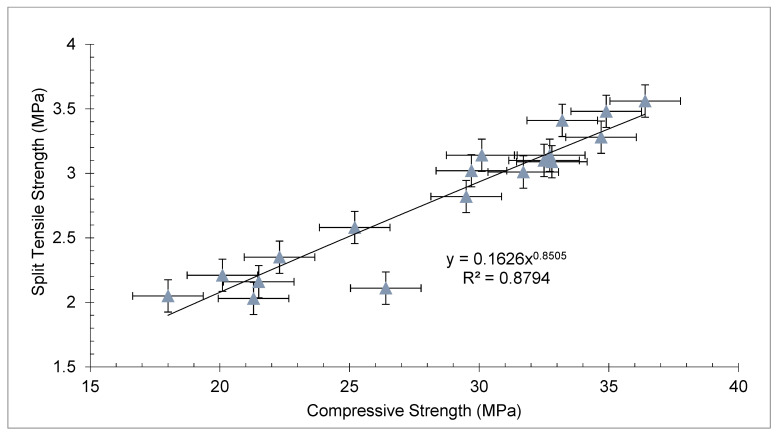
Correlation between compressive strength and split tensile strength.

As previously stated, the split tensile strength follows the same pattern as the compressive strength. Split tensile strength is 10–15% of concrete’s compressive strength. As a consequence, compressive and split strength correlated significantly. Figure 8 shows a regression model with an R^2^ of approximately equal to 90%.

The following equation has been developed with different doses of coconut fibers:

f_sp_ = 0.16 × fc^0.85^ (1)

where,

f_sp_ = Split tensile strength_,_ f_c_ = Compressive strength

However, different codes recommended different equation to predict split tensile strength from compressive strength, which are listed below. ACI-318.11 [58] Equation (2), Eurocode [59] Equation (3), and JSCE-07 [60] Equation (4).

(2) $$f_{\mathrm{sp}}=0.53\;\times \;\;\sqrt{fc}$$

(3) $$f_{\mathrm{sp}}=0.3\;\times \;\sqrt{fc}$$

(4) $$f_{\mathrm{sp}}=0.44\;\times \;\sqrt{fc}$$

**Figure 9:**
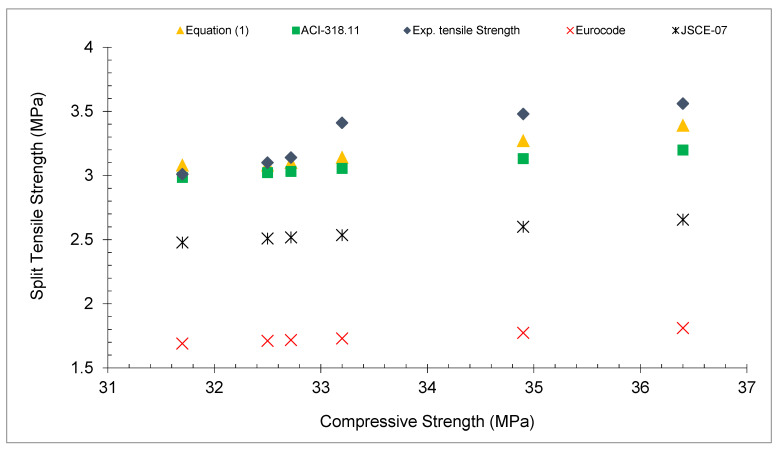
Experimental and predicted tensile strength.

A comparison among split tensile strength from Equation (1), different codes, and past studies is given in Figure 9 and Table 3. It may be noted that the experimental tensile strength from paste studies, predicted from Equation (1) and ACI-318.11 [58], approximately gives the same results. However, the code Eurocode [59] and JSCE-07 [60] give results that are less than the experimental results of the past study. The results with various amounts of fiber are more scattered than the predicted tensile strength from Eurocode [59] and JSCE-07 [60]. These codes were applied to conventional concrete. The results found by previous findings with different doses of coconut fiber also did not follow these codes except ACI-318.11 [58]. However, the equation developed in the study with different doses of coconut fibers was much nearer to the tensile strength of the previous research (experimental results). Instead of using codes for concrete with varying amounts of coconut fibers, this study’s equation yielded more accurate results.

### 5.3. Flexure Strength 

The tensile capacity of concrete may be determined in many ways, one of which is a flexural test. Flexure strength is the capability of an unreinforced concrete beam or reinforced concrete beam to withstand bending failure. According to ASTM standards, it is determined by loading 6 × 6-inch (150 × 150-mm) concrete beams with a span length that is at least three times the depth [61].

**Figure 10:**
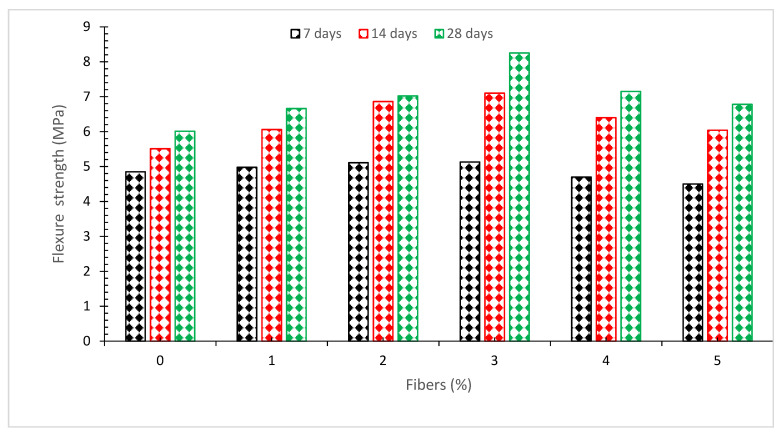
Flexure strength [24].

Figure 10 shows the flexure capacity of concrete with various quantities of coconut fiber, from 0% to 5% in 1% increments. The flexure strength of concrete rose to 3% with the addition of coconut fiber and then steadily reduced, with the lowest flexure strength happening at 0% and the greatest occurring at 3%. Additional research found that when the percentage of coconut fiber is raised by the weight of cement by up to 2.0 percent, the flexure strength improves, but subsequently drops when the percentage of coconut fiber is further increased as compared to a reference or standard concrete [62]. The inclusion of coconut fibers at a rate of 2.0 percent resulted in the greatest possible flexure strength. After the addition of coconut fiber at a rate of beyond 2.0 percent, the flexure strength gradually reduced. The flexural strength of all coconut fiber reinforced concrete is much higher than that of ordinary concrete. Coconut fibers increase flexural capacity by inhibiting the development of fractures. Because of the interfacial between the concrete components and the coconut fibers, the load is quickly transmitted to the coconut fibers. The breaking of cracks is prevented by coconut fibers, which allow the crack to flow around the fibers and transfer the stress. The coconut fibers and concrete matrix resist the load as a whole, resulting in increased flexure strength for the structure [49]. The compaction procedure becomes more complicated as the quantity of fiber in the mixture rises. Using higher dosages, such as 3.0 percent, the workability of the concrete was worsened, causing porous concrete and a drop in flexural strength. According to other findings, the best quantity of CF in concrete is 0.25 percent, which results in a 19 percent increase in 28-day flexural capacity [39]. Table 4 shows a summary of the flexural strength of concrete with different doses of coconut fibers.

**Figure 11:**
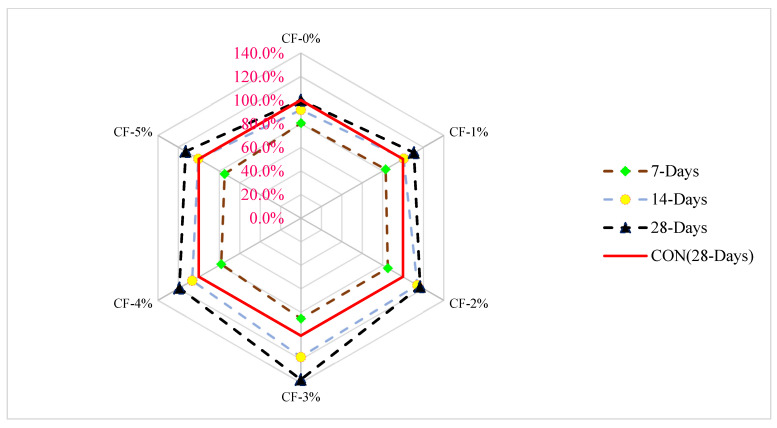
Relative analysis of flexure strength [24].

Figure 11 shows a relative analysis of flexure strength with a differing dose of coconut fiber at various days of curing. The flexure strength of reference concrete at 28 days of curing was considered as a reference mix, from which the flexure strength of other doses of coconut fiber (CF) was compared at various days of curing. The optimum dose of CF (3%) was considered for comparison analysis. At 7 days of curing, flexure strength is 15% lower than that associated with control flexure strength (28 days of control concrete flexure strength at 3% addition of CF. At 3% addition of CF, the flexure strength is 18% more than the reference concrete compressive strength at 14 days of curing. At a similar dose of CF (3%), flexure strength is 38% more than the reference concrete. It is worth mentioning that coconut fiber improved flexure strength (47%) more effectively than compressive strength (12%). A study also reported that the fibers enhanced flexure capacity more efficiently than compressive capacity at the same dose and same dose of curing [26].

**Figure 12:**
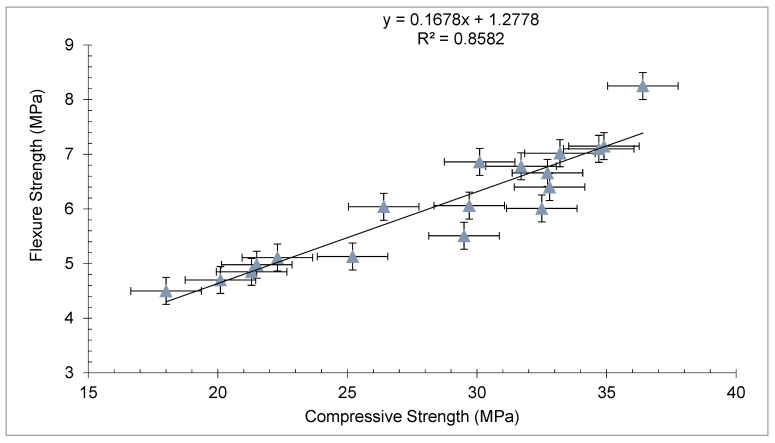
Correlation between compressive strength and flexure strength.

A regression model between compressive and flexural strength is shown in Figure 12. It can be observed that a strong correlation exists between compressive and flexural strength, with the R square approximately equal to 90%.

## 6. Durability 

### 6.1. Water Absorption 

The water absorption test analyses the rate of water absorption of the outer and inner concrete surfaces. The test includes measuring the increase in mass of concrete samples caused by water absorption as a function of the time when the specimen is exposed to water. Higher water absorption results in less durability since water contains various hazardous compounds that seep into the concrete, causing concrete breakdown and resulting in reduced durability.

**Figure 13:**
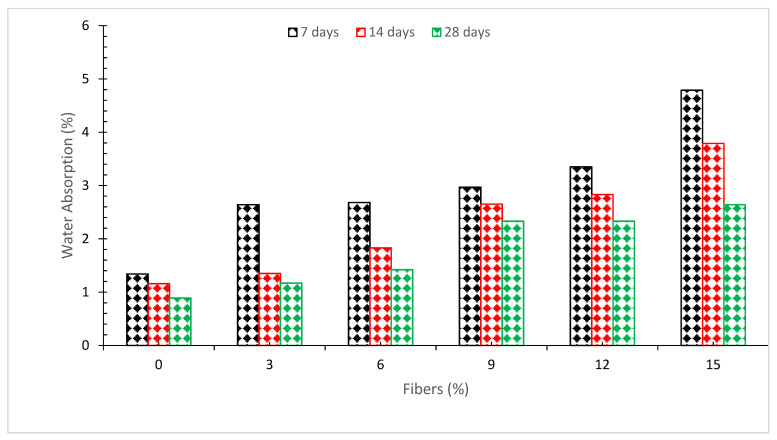
Water absorption [73].

Figure 13 depicts the water absorption of concrete with various doses of coconut fiber. With the addition of coconut fibers, it can be noted that the amount of water absorbed increased [73]. Research revealed that the effects of fiber volume fraction on heat conductivity and water absorption were not significant [68]. A study concluded that the water absorption decreases as the percentage of coconut fibers increase up to 2.0 percent addition of coconut fibers, and the decrease occurs gradually, with maximum water absorption observed at 0 percent substitution and minimum water absorption observed at 2.0 percent addition of coconut fibers [15]. It has also been reported that minimal water absorption was achieved when steel fibers were added at a rate of 2.0 percent [38]. This is due to the fact that the elastic modules of conventional concrete are lower than those of fiber-reinforced concrete. The inclusion of CF would result in an increase in the tensile strain characteristics of concrete, which would limit the creation and development of early fractures in the concrete [74]. In other words, increasing concrete density reduces water absorption. Due to lack of workability, greater dosages (above 2.0%) resulted in less dense concrete. Because of the increased porosity of the coconut coir fraction mortar compared to the control mortar, according to one research, more water absorption was noticed in coconut fiber reinforced concrete than in the control mortar. The porous structure of the cement blocks, as well as the presence of an interfacial zone surrounding the particles, are the most important elements influencing water absorption. The findings reveal that as compared to the control mortar, the coconut coir mortar noticed water absorption considerably in the greater amount [25].

### 6.2. Carbonation Depth 

**Figure 14:**
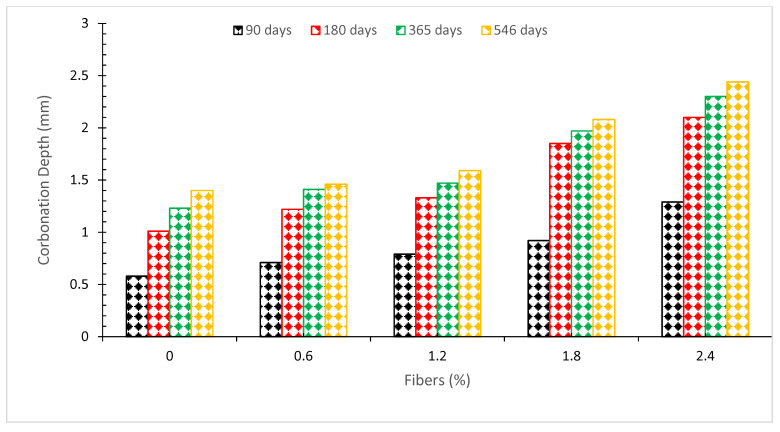
Carbonation depth [37].

The carbonation of concrete is a crucial characteristic connected with steel reinforcement corrosion. Carbonation depth entirely depends on permeability. By contrast, other influencing variables such as temperature, carbon dioxide concentration, water/binder ratio, and relative humidity are consistent throughout all specimens. The findings show that carbonation depth rises with fiber concentration, implying that carbonation happens faster on a more porous material. The 2.4 percent CF carbonation depth was 74.3 percent greater than the reference concrete at 546 days, while the 0.6 percent CF result was equivalent to the reference concrete with a 4.3 percent difference as shown in Figure 14. The research also found that the carbonation depth of concrete reduces as the number of CF increases from 0% to 2.0% by weight of cement. Conversely, all the coconut fiber reinforced concrete has a lower carbonation depth than normal concrete. The minimal depth was observed at a 2.0% addition of CF, which is over 48% (after 14 days) lower than conventional concrete [15]. Many holes are developed in concrete as a result of the loss of accessible water and shrinkage, which makes it easier for CO_2_ to diffuse through the concrete. The addition of coconut fibers (CF) limits the CO_2_ diffusion channel, resulting in an increase in the resistance to CO_2_ penetration into the concrete. As a consequence, the rate of carbonation depth has decreased significantly [75]. In contrast, when using a higher dosage of CF (3.0 percent), the fiber inhibits the cement paste from filling the voids in the microstructure, resulting in increased internal porosity. In addition, the fiber can create a new channel for CO_2_ to penetrate the concrete, resulting in enhanced carbonation speed in the concrete structure [42].

### 6.3. Permeability 

The water absorption by immersion provides an estimate of the total (reachable) pore volume of the concrete, but it provides no information on the permeability of the concrete, which is more essential in terms of long-term performance.

**Figure 15:**
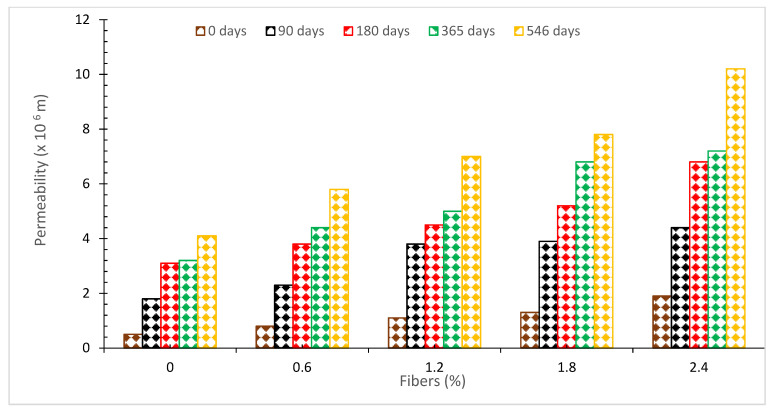
Permeability [37].

The permeability of concrete after the addition of coconut fiber is seen in Figure 15. It was discovered that the permeability of concrete increased with the addition of coconut fibers. The continuous pore structure of the specimens has a significant influence on the permeability of the specimens [76]. Generally speaking, the wider the width of continuous pores, the more permeable the concrete would be considered to be. It is possible to produce continuous holes under a variety of situations, some of which include the capillary network formed by hydration, the interfacial transition zone between paste and aggregate, the production of micro-cracks, and others [77]. Not only the interfacial zones between the aggregate and the paste but there will also be a gap between the interfacial zones between the fiber and the matrix [78]. However, the authors hypothesized that there is another mechanism at work that is also responsible for the disparity. This gap may be linked to the strong water absorption characteristics of the coconut fiber, which is responsible for its existence. During the mixing process, a water film is formed around the fiber’s immediate surroundings. The absorption capabilities of the film, as well as the osmosis pressure, will keep the film in place and cause the fiber to inflate. As the hydration process advances, the formation of the permanent shell structure starts to take place. As water is consumed by cement hydration and evaporation, the water film gradually vanishes, finally creating a gap between the fiber/matrix interfacial zones of the composite. When the fiber shrinks back to its former shape after inflating as a result of the drying process, the gap widens even further, creating a larger opening. According to the findings of a study, nitrogen gas travels through the natural fiber during the permeability test because the natural fiber has a porous cellular structure, increasing the likelihood of pore network connection during the test [79].

## 7. Scan Electronic Microscopy (SEM)

**Figure 16:**
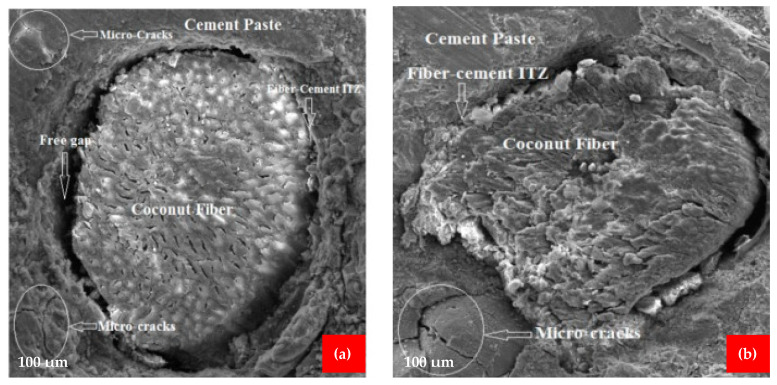
SEM of (**a**) free space between cement paste ITZ and micro cracks, (**b**) micro cracks, and CF paste [50].

**Figure 17:**
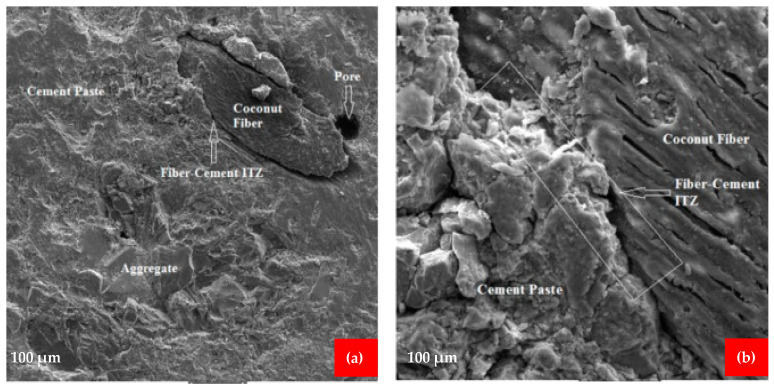
(**a**) Pore in ITZ, (**b**) CF paste ITZ [50].

**Figure 18:**
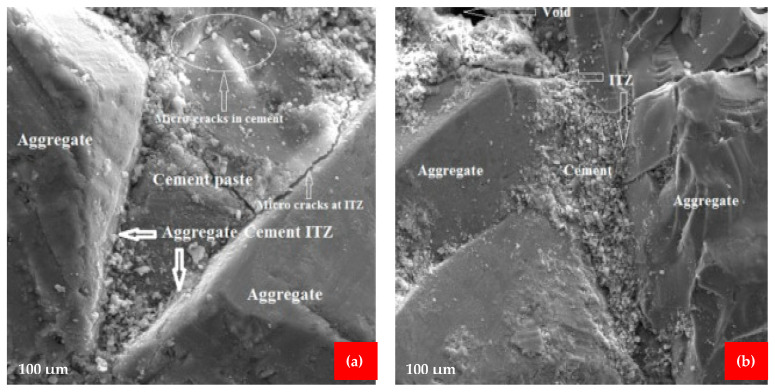
(**a**) Cracks in paste and aggregates, (**b**) aggregates paste ITZ [50].

The use of scanning electron microscopy (SEM) improves the capacity to evaluate the microstructure of cement and concrete. It will also assist in analyzing the impacts of supplemental cementing ingredients or fibers, as well as evaluating concrete durability issues. The microstructure of the coconut fiber reinforced concrete was investigated using SEM [50]. The samples for SEM were prepared from the coconut fiber reinforced concrete beams after flexure testing. The goal of the SEM examination was to investigate the interfacial transition zones (ITZs) between the fiber-cement paste and aggregate-cement paste, as well as micro-cracks and their propagation through the matrix. As shown in Figure 16a,b, free space was detected at the ITZ between the fiber and the cement paste, indicating that the fiber had a poor bond with the cement paste. It is due to the fact that coconut fiber reinforced concrete samples have a lower modulus of rupture than high-strength concrete. According to Figure 17a,b, a suitable bond between the fiber and cement paste was formed, i.e., there were no gaps between the fiber and cement paste, resulting in enhanced mechanical qualities. The microcracks in the cement paste and aggregates are seen in Figure 16a,b, and Figure 18a. Crack propagation was detected across both the aggregate and the cement paste, indicating a strong fiber-cement paste ITZ and an enhanced microstructure. The addition of silica fume to the matrix resulted in an improvement in the microstructure of the matrix due to the strong pozzolanic activity of silica fume. This indicates that upon hydration in an alkaline environment, calcium silicate hydrates (CSH) gel is created because of silica-fume interacting with calcium hydro oxide, which is formed during the hydration of cement. The CSH gel decreases the porosity of the matrix by filling the pores, thus resulting in an increase in the strength of the concrete. The silica fume cannot hydrate directly with water, it combines with Ca(OH)_2_ to form CSH. Ca(OH)_2_ may also have an unfavorable influence on the interfacial and microstructure characteristics of a material owing to the orientation and crystallinity of the material. A study also reported that Ca(OH)_2_ is chemically active, which causes a decrease in the durability of concrete by reacting with other chemicals, forming harmful compounds [80]. The inclusion of silica fume may increase the overall matrix strength to a certain amount. However, the addition of fibers in greater numbers might result in lower strengths owing to non-uniformity due to a lack of flowability [81]. A study also reported that pozzolanic materials improved the microstructure characteristics of concrete due to micro filling, which gives a more compact mass [82]. The incorporation of coconut fiber into the concrete resulted in the formation of new interfaces within the matrix, which resulted in weak linkages between the coconut fiber and the matrix. There were open spaces created because of the debonding of the coconut fiber from the matrix (Figure 16a). The increase of coconut fiber in higher amounts weakens the binding strength of the matrix because of the lower flowability. The creation of the free space may also be caused by fiber breaks in the surrounding tissue. Because of the existence of this free gap, the post-cracking performance of the matrix material was reduced. Figure 17b illustrates how a good link between coconut fibers and the cement matrix should be free of gaps, resulting in improved mechanical characteristics for the coconut fibers. It is also significant to note that the slippage of fibers in relation to the matrix is the primary cause of the increased strength of concrete. The fibers are surrounded by cement paste inside the matrix, which increases the strength of the matrix as a result of a stress transfer mechanism between the matrix and the reinforced fibers. Furthermore, to a certain degree, the tensile stresses created by the application of a load are also resisted by fibers, which contributes to the preservation of the material by preventing crack propagation from occurring.

## 8. Conclusions 

This paper presents a summary of research progress on natural fibers (coconut fibers). Coconut fibers are an inexpensive, recyclable, low-density, and environmentally acceptable building material. These fibers have excellent tensile qualities, and they may be utilized instead of traditional fibers such as glass and carbon steel fibers. Based on a detailed review, the following conclusions have been made:

- The flowability of concrete decreased with the addition of coconut fiber due to the larger surface area of the fiber, which enhanced the internal friction among concrete ingredients, leading to less workability. Furthermore, an increase in fresh density is observed up to 2% addition of coconut fibers.
- Mechanical characteristics such as compressive, split tensile, and flexure strength were improved up to a certain dose of coconut fiber, which depends on physical properties of fibers such as length, diameter, and aspect ratio. Furthermore, it can also be observed that coconut fibers improved flexure capacity (47%) more efficiently than compressive capacity (12%).
- Increased durability properties were also observed with the addition of coconut fibers. However, less information is available in this regard.
- The optimum dose of coconut fibers is the most important parameter for better performance of concrete, as a higher dose results in more voids in hardened concrete due to lack of workability, leading to lower mechanical and durability performance of concrete. The optimum dose of concrete varies depending on fiber length, diameter, and aspect ratio. However, the majority of researchers recommended the optimum dose of coconut fiber is from 2 to 3% by volume of cement.

It can be concluded that coconut fibers enhanced flexure capacity more efficiently than compressive capacity. Therefore, further research was recommended to add some pozzolanic materials such as fly ash and silica fume to the improved compressive capacity of fiber reinforced concrete for high strength concrete. It is necessary to conduct detailed research investigations into the influence of coconut fibers on crack abridgment, cement matrix pore structure, water, and chloride permeability properties of concrete. It is also necessary to investigate a novel strategy that makes use of the water retention capacity of coconut fibers in order to generate high-performance cement composites using internal curing technology. 

## Figures and Tables

**Table 1:** Physical properties of coconut fibers.

Property Name	Naveen et al. [33]	Amadi et al. [34]	Bai et al. [35]	Ramakrishna et al. [36]	Ahmad et al. [15]	Ramli et al. [37]
Length (mm)	-	25	18	60 to 250	8 to 10	20 to 30
Diameter (mm)	-	0.25	0.1 to 0.5	0.40 to 0.10	0.5 to 1.0	0.32
Aspect Ratio	-	100	-	-	-	-
Tensile Strength (MPa)	175	405	-	15 to 327	-	176
Modulus (GPa)	4 to 6	4	-	-	4.5	22.4
Density (g/cm^3^)	-	-	0.67 to 10	-	-	-
Water Absorption (%)	130 to 180	-	-	-	-	-
Elongation (%)	30	-	-	75	25	-

**Table 2:** Treatment of CF [44].

Coating	Solution	Details
Silica fume	Water	Coconut fibers were immersed in a deionized water solution for 60 min while being constantly agitated. Following that, the fibers were put in a receiver that was filled with silica fume to dry. The coconut fiber began to get covered with a thin coating of silica fume on its surface as the process progressed.
Silica fume	Latex	The same silica water treatment technique is followed, with the exception that the deionized water solution is replaced with a natural latex solution at a concentration of 1 percent.
Metakaolin	Water	The identical silica water treatment process is applied, with the exception that silica fume is used in place of metakaolin instead of the latter.
Metakaolin	Latex	The metakaolin water treatment technique is the same as before, with the exception that metakaolin is used instead of silica fume.
Nil	Nil	Using natural coconut fiber (that has not been treated) and incorporating it into the cementitious matrix for the fabrication of specimens for the durability test

**Table 3:** Experimental and predicted tensile strength.

Experimental Compressive Strength (MPa)	Equation (1)	ACI-318.11 [58]	Eurocode [59]	JSCE-07 [60]	Experimental Split Tensile Strength (MPa)
32.5	3.08	3.02	1.71	2.50	3.1
32.72	3.10	3.03	1.71	2.51	3.14
33.2	3.14	3.05	1.72	2.53	3.41
36.4	3.39	3.19	1.80	2.65	3.56
34.9	3.27	3.13	1.77	2.59	3.48
31.7	3.08	2.98	1.68	2.47	3.01

**Table 4:** Summary of mechanical performance of concrete with coconut fibers.

Author/ Reference	Percentage of Replacement	Compression Strength (MPa)	Flexure Strength (MPa)	Split Tensile Strength (MPa)
Abbass et al. [63]	0 0.1 0.2 0.3 0.4 0.5 0.6	36 38 38 36.5 35 33.5 30	4.8 5.2 5.4 4.98 4.85 4.75 4.50	3.61 3.70 3.90 3.85 3.60 3.50 3.30
Srinivas et al. [64]	0 0.5 1 1.5	8.0 8.66 9.93 4.75	6.33 3.23 3.82 2.80	2.95 0.87 0.95 0.92
Kumar et al. [16]	0 CF%:5 CF ASH%:15	22.3 19.53 34.87	6.73 5.27 5.33	1.28 2.39 1.378
Khan et al. [65]	Silica Fume:CF 0:2 5:2 10: 2 15: 2 20: 2	27.2 27.5 28.8 32.4 26.6	6.2 6.6 7.8 8.3 4.7	3.0 3.4 3.5 3.6 2.9
Das et al. [66]	Steel fiber:CF 0:0 1:2 1:4 1:6	19.26 20.42 18.58 17.66	3.94 492 4.02 3.56	3.62 4.32 3.76 2.98
Raj et al. [67]	0 0.3 0.4 0.5	9.5 11.5 8.0 7.5	1.4 1.7 1.2 1.1	3.50 3.48 2.45 2.10
Wongsa et al. [68]	0 0.5 0.75 1	31 33 28 25	3.2 5.3 6.2 6.7	1.8 2.1 2.2 2.4
Krishna et al. [69]	0 0.5 1 1.5 2	37.5 35 47.5 51 41.75	-	-
Sathiparan et al. [25]	0.000 0.125 0.250 0.500 0.750	2.8 2.83 2.86 2.75 2.62	1 1.12 1.14 1.15 0.84	-
Hwang et al. [70]	0 1 2.5 4	65 50 48 40	5.2 5.5 6.4 7.5	-
Korniejenko et al. [71]	Control Coir fibers (1%) Cotton fibers (1%) Raffia fibers (1%) Sisal fibers (1%)	24.78 31.36 28.42 13.66 25.16	5.55 5.25 5.85 3.05 5.90	-
Ali et al. [40]	Length (2.5 cm:5 cm:7.5 cm) 1:1:1 2: 2:2 3: 3:3 5: 5:5	42:43.5:37 41:42.5:33.5 40:38:31.5 36.5:36:00	-	3.85:4.3:4.35 3.80:4.30:4.20 3.35:4.25:4.00 3.5:3.75:0.00
Baruah et al. [72]	0 0.5 1.0 1.5 2.0	21.42 21.70 22.74 25.10 24.35	-	2.88 3.02 3.18 3.37 3.54

## Data Availability

All the data available in the manuscript.
